# Atomic Layer Deposition for Zinc-Ion Batteries: Nanoarchitectonics for Next-Generation Energy Solution

**DOI:** 10.1021/accountsmr.5c00326

**Published:** 2026-06-25

**Authors:** Shaista Nouseen, Sujit Deshmukh, Martin Pumera

**Affiliations:** † Quantum Materials Laboratory, 3D Printing & Innovation Hub, Center for Nanorobotics and Machine Intelligence, Department of Chemistry and Biochemistry, Mendel University in Brno, Zemedelska 1, Brno 61300, Czech Republic; ‡ Future Energy and Innovation Laboratory, Central European Institute of Technology, 48274 Brno University of Technology, Purkynova 123, Brno 61200, Czech Republic; § Department of Condensed Matter and Materials Physics, S. N. Bose National Centre for Basic Sciences, Block JD, Sector III, Salt Lake, Kolkata 700106, India; ∥ Advanced Nanorobots and Multiscale Robotics Laboratory, Faculty of Electrical Engineering and Computer Science, VSB - Technical University of Ostrava, 17. listopadu 2172/15, Ostrava 70800, Czech Republic; ⊥ Department of Chemical and Biomolecular Engineering, Yonsei University, 50 Yonsei-ro, Seodaemun-gu, Seoul, 03722, Korea

## Abstract

With the existing boom in the requirement for energy storage devices for a broad range of applications, ranging from portable to wearable devices, different types of batteries such as zinc-ion batteries (ZIBs), lithium-ion batteries (LIBs), sodium-ion batteries, aluminum-ion batteries, etc., have been currently explored. Among them, recently ZIBs have been extensively studied as an alternative to LIBs because of their inherent safety, high theoretical capacity, low cost, and sustainable approaches. Nevertheless, several limitations impede their practical applications, which include Zn dendrite growth and corrosion on the anode side, side reactions, cathode dissolution, etc. To address these concerns, enormous efforts have been under investigation, such as developing novel cathode materials with customized electrode design, integrating different nanoengineering techniques and composite materials, anticipated to achieve excellent ZIB electrochemical performance. In this context, atomic layer deposition (ALD) technique is garnering significant attention, offering several benefits like a meticulous, scalable, and sustainable approach, with great potential in generating protective nanoengineered layers on the surface of the anode and the cathode components of ZIBs with conformal film thickness and atomic-level precision, which is extremely difficult to achieve with other existing techniques, such as chemical vapor deposition and physical vapor deposition.

In this Account, we highlight the evolution and progress of the ALD technique, where ALD-modified electrodes are employed for electrochemical energy conversion and storage applications. First, we provide a brief introduction of the ZIBs as a sustainable energy storage solution and ALD technology as an innovative technique to modify the anode and the cathode surface of electrodes. Furthermore, the fundamental electrochemistry of ZIB, including the challenges faced on the cathode and anode surface of electrodes, is discussed; in addition, the fundamental chemistry of the ALD technique and its mechanism are discussed. Next, the crucial role of the ALD technique to modify cathode and anode surfaces, to tackle several ZIBs’ challenges including cathode dissolution, Zn anode corrosion, and dendrite growth, is briefly discussed. Finally, we attempt to conclude that ALD is a viable solution to resolve the challenges associated with ZIBs and provide a perspective on the future research direction of the ALD technology for ZIBs.

## Graphical abstract

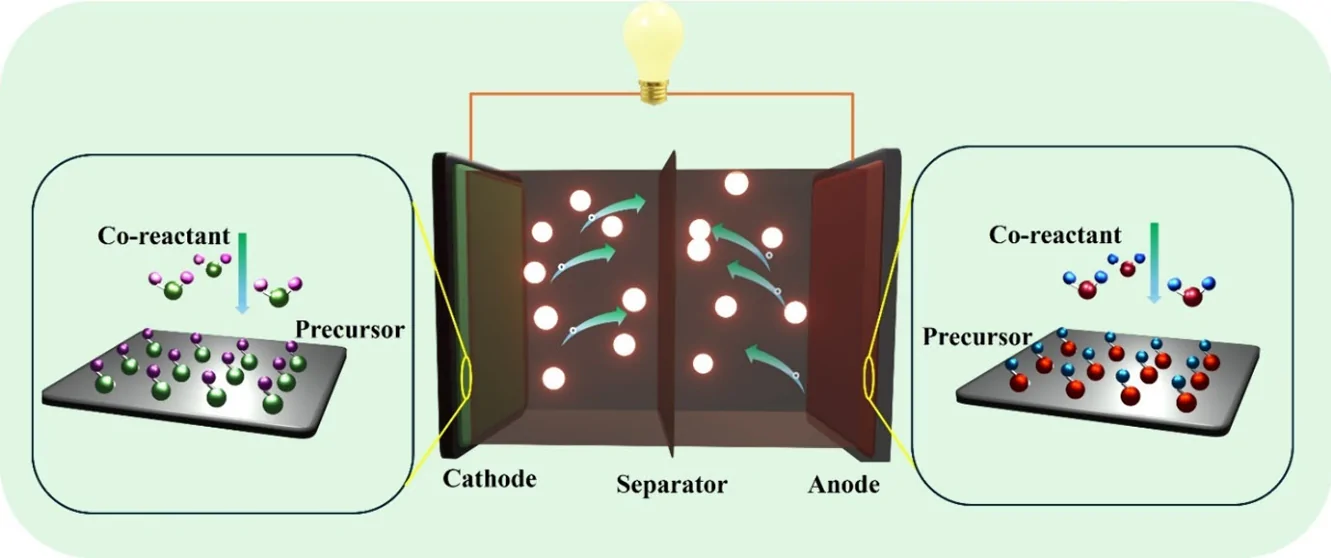

## 1 Introduction

With the current rise in the technology industry and improving living standards of human society, the demand for affordable and clean energy storage modules with exceptional energy/power density and long cyclic stability has increased exponentially, especially in the wearable/portable electronic and electric vehicle sectors.^1,2^ For years, LIBs have been regarded as one of the advanced electrochemical energy storage solutions and have dominated the leading commercial energy storage market since the 1990s. Nonetheless, inadequate capacity output of insertion-type oxide cathodes and commercial graphite anodes, increased level of safety issues, environmental contamination, and high cost make researchers think beyond LIBs for a sustainable future.^1–4^

In recent times, the aqueous electrolyte-based ZIBs have been regarded as one of the promising alternatives beyond LIBs, in terms of both economic and ecological aspects. Note that the ionic conductivity of aqueous-based electrolytes (∼1 S cm^–1^) is superior to that of nonaqueous organic electrolytes (∼10^–2^ to 10^–3^ S cm^–1^).^5^ Aqueous electrolytes are thermodynamically limited to a ∼1.23 V stability window due to water splitting. The hydrogen evolution potential implies that metals with more negative redox potentials, such as Li (−3.04 V vs SHE) and Mg (−2.38 V vs SHE), are unstable as anodes in water.^6^ However, Zn metal exhibits a high overpotential for hydrogen evolution, effectively suppressing H_2_ formation.^6^ This kinetic barrier enables Zn to function as a stable metal anode in aqueous systems despite thermodynamic constraints. Zinc metal’s relatively low redox potential (−0.76 V vs SHE) in aqueous electrolytes enables obtaining high cell voltage and exceptional theoretical capacity up to ∼820 mAh g^–1^ or ∼5854 mAh cm^–3^.^7–10^

Regardless of their promising attributes, there are major challenges in ZIBs. The uneven Zn electrodeposition due to the nonuniform Zn^2+^ ion flux causes the formation of lumps over the anode surface that intensify local electric fields (“tip effect”) and grow into dendrites during cycling.^11^ These dendrites can cause rapid decay of Coulombic efficiency (CE) and battery capacity and may eventually pierce the separator, causing short circuits and battery failure.^12,13^ Another major limitation is the Zn metal corrosion due to the H_2_ evolution. We already discussed that a relatively high overpotential of the Zn metal against H_2_ evolution reduces the reaction rate, but a low rate still constantly corrodes the Zn. To be precise, the enhancement of OH^–^ concentration causes the formation of byproducts like Zn_4_SO_4_(OH)_6_·*x*H_2_O, which cause sluggish Zn^2+^ ion diffusion across the interphase.^14^ These side reactions continuously consume Zn and the electrolyte, limiting the shelf life of ZIBs.^15^ In addition, cathode materials also commonly face issues in the ZIB module such as the dissolutions of transition metal ions and structural degradation during the cycling process.^16,17^ Interface decoration or coating to alter the anode or cathode’s surface is a promising strategy to suppress dendrite formation, enhance interfacial stability by preventing direct contact between H_2_O and the electrode, and reduce byproduct formation from side reactions.

In this context, different deposition procedures, such as chemical vapor deposition (CVD), physical vapor deposition (PVD), and atomic layer deposition (ALD), could be potentially explored to modify the anode and cathode surfaces of the battery system. However, CVD and PVD are bulk deposition methods and need high-temperature conditions compared to ALD.^18–23^ ALD is superior, as it is a surface-controlled process with customized conformal deposition of materials and controlled atomic-level thickness onto the complex architectures occurring at lower temperature, which is crucial for electrochemical energy storage applications,^18–23^ as the interfaces and surface properties tend to significantly influence the stability and ion transport kinetics of the materials. Therefore, ALD technology could be useful to tailor the electrodes, boosting their stability, generating a protective layer, improving the electrode–electrolyte compatibility, and introducing functional interlayers to boost the overall ZIB electrochemical performance.^14,23–25^

Though ALD has been extensively applied to improve the electrochemical performance of multiple electrochemical energy applications like Li- and Na-based battery performance,^24,25^ catechol oxidation,^26^ electrocatalysis and photo-electrocatalysis,^27–30^ nitrate conversion reactions,^31,32^ and supercapacitors,^33^ its potential in aqueous ZIBs remains largely underexplored. In this Account, we primarily focused on providing comprehensive insight into ALD technology utilizing different precursor materials for zinc-ion battery applications and concisely describe the evolution of ALD technology for the expansion of ZIB technology. This Account briefly covers the fundamental electrochemistry of aqueous ZIBs, anode and cathode reactions and their key limitations, the principles of ALD technology, its potential role in improving ZIB performance, and future perspectives. The limitations of ZIBs at both the anode and cathode are comprehensively highlighted along with a detailed discussion on how ALD technology can be used to address these challenges. In future directions, the possibility of innovation to design novel precursor materials with a combination of different technologies is briefly discussed.

## 2 Fundamental Electrochemistry of Aqueous Zinc-Ion Batteries

ZIBs are made up of various components, which include the cathode, anode, separator, current collector, and electrolytes.

### 2.1 Anode Reactions

In alkaline and neutral environments, the zinc anode operates through two different reactions. For example, in an alkaline solution, zinc undergoes oxidation during discharge by releasing two electrons and interacting with hydroxide (OH^–^) ions present in the electrolyte, producing Zn­(OH)_4_^2–^. At a certain point, the concentration of Zn­(OH)_4_^2–^ reaches saturation, and it naturally breaks down into solid ZnO, which accumulates over time on the Zn anode surface. During charging, the Zn­(OH)_4_^2–^ species gains electrons and converts back into metallic zinc. This reversible electrochemical reaction of the zinc anode in alkaline conditions can be described by the following reactions.^34^

Discharge:

$$\begin{array}{l} Zn+4OH^{-}\to Zn{\left(OH\right)}_{4}^{2-}+2e^{-}\\ Zn{\left(OH\right)}_{4}^{2-}\to ZnO+2OH^{-}+H_{2}O \end{array}$$

Charge:

$$\begin{array}{l} ZnO+2OH^{-}+H_{2}O\to Zn{\left(OH\right)}_{4}^{2-}\\ Zn{\left(OH\right)}_{4}^{2-}+2e^{-}\to Zn+4OH^{-} \end{array}$$

In a neutral pH, the zinc anode participates in reversible plating and stripping processes, where Zn^2+^ ions act as the primary charge carriers. During the process, zinc alternately deposits on and dissolves from the anode surface. The detailed reaction mechanism is outlined below:

Discharge:

$$Zn\to Zn^{2+}+2e^{-}$$

Charge:

$$Zn^{2+}+2e^{-}\to Zn$$

Note that the reduction potential of Zn/ZnO in an alkaline electrolyte is −1.22 V against SHE, whereas for a neutral electrolyte the value is −0.76 V SHE.^34^

### 2.2 Cathode Reactions

The cathode reactions associated with Zn^2+^ ions can generally be categorized into three parts: (1) insertion and extraction of Zn^2+^ ions (Figure 1a), (2) co-insertion and removal of both H^+^ and Zn^2+^ ions (Figure 1b), and (3) electrochemical conversion reactions (Figure 1c).^34^

**Figure 1:**
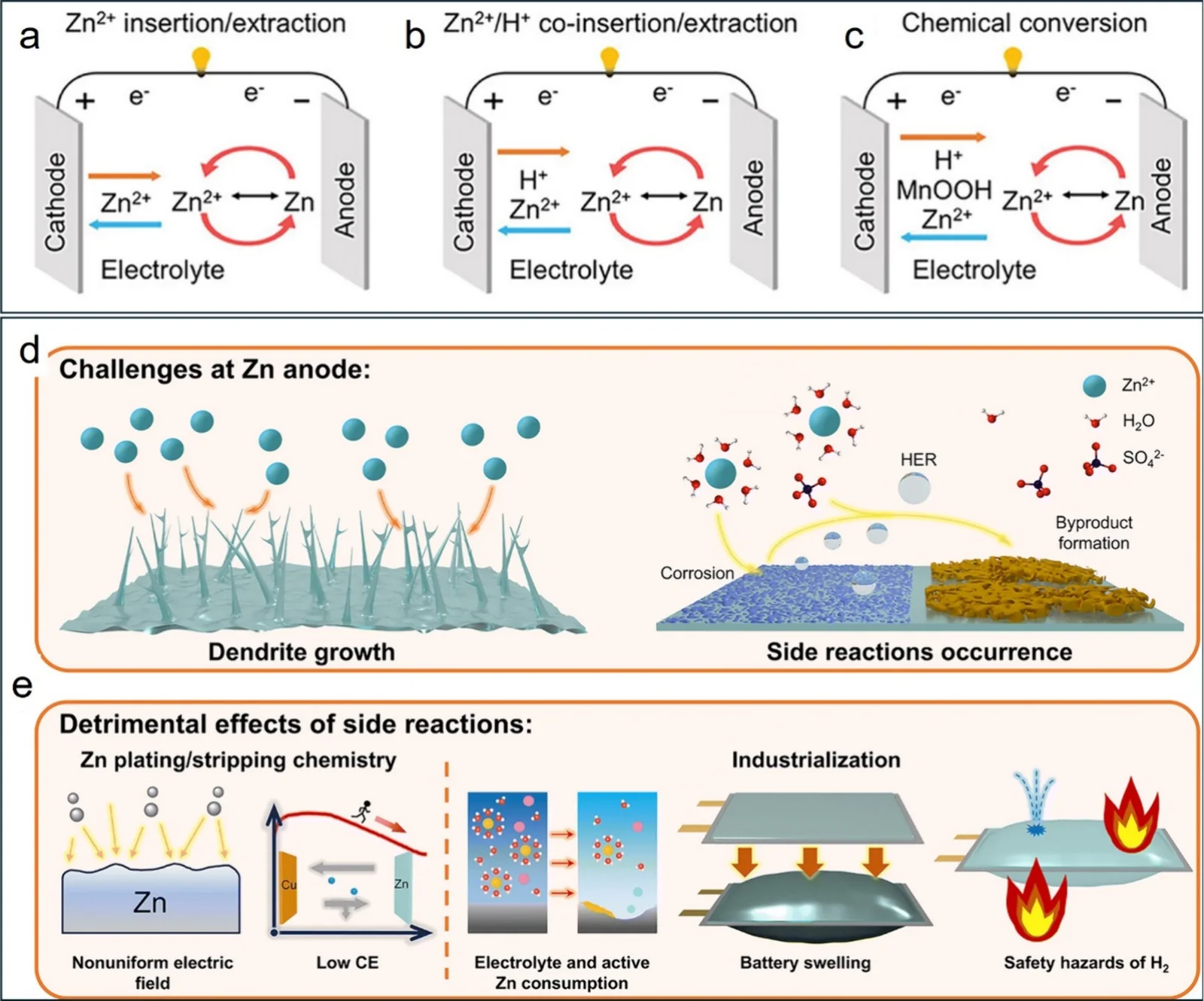
(a–c) The basic charge storage reaction mechanism for aqueous zinc-ion batteries. Reproduced with permission from ref 34. Copyright 2026 Wiley-VCH under Creative Commons Attribution 4.0 International CC BY 4. (d, e) Major limitations of ZIBs at the anode side and detrimental effects of side reactions. Reproduced with permission from ref 12. Copyright 2026 Royal Society of Chemistry under Creative Commons Attribution NonCommercial 3.0 Unported License.

In 2012, Xu et al. first identified Zn^2+^ intercalation/deintercalation in a tunnel-structured α-MnO_2_ cathode in aqueous electrolytes (Zn­(NO_3_)_2_ or ZnSO_4_).^35^ Studies showed that Zn^2+^ insertion alters the α-MnO_2_ phase to a layered birnessite phase, with Zn^2+^ storage strongly influenced by MnO_2_ crystal forms (α, β, γ, δ).^36^ Comparable mechanisms are also observed in layered vanadium compounds and Prussian blue analogs.^37,38^

Next, the H^+^/Zn^2+^ co-insertion mechanism, where the small radius and fast-diffusing H^+^ enhance active site utilization, is studied. In Zn||MnO_2_ systems, H^+^ inserts first during discharge, followed by Zn^2+^.^39^ In the conversion-type reaction, α-MnO_2_ can transform MnO_2_ and MnOOH. This mechanism involves the formation of MnOOH and ZnMn_2_O_4_ upon discharge, along with the accumulation of byproducts (like Zn_4_(OH)_6_SO_4_·*n*H_2_O) on the cathode surfaces due to water decomposition.^40^

## 3 Challenges of Aqueous Zinc-Ion Batteries

Regardless of the ZIB benefits, which we discussed earlier, different challenges persist that impede their practical applications and their long-term electrochemical performance. Some of these challenges are summarized below:

### 3.1 Challenges of the Anode Side

The major issues on the anode side mainly fall into two categories: dendrite formation and parasitic side reactions, such as hydrogen evolution (HER), corrosion, and byproduct generation. Irregular zinc deposition leads to dendritic structures that can penetrate the separator and trigger short circuits (Figure 1d and e).^12^ At the same time, zinc’s thermodynamic instability in aqueous media promotes HER and corrosion, which consume electrolyte, produce gas bubbles, and corrode the anode surface. The HER primarily occurs because the reduction potential of Zn^2+^/Zn is −0.76 V vs SHE, which is lower than the H_2_O/H_2_, resulting in a reaction between Zn metal and H_2_O, which releases H_2_ gas.^41^

$$\begin{array}{l} \mathrm{Anode:}\;Zn\to Zn^{2+}+2e^{-}\\ \mathrm{Cathode:}\;2H_{2}O+2e^{-}\to 2OH^{-}+H_{2}↑ \end{array}$$

As hydrogen gas builds up over time, the rising internal pressure may lead to electrolyte leakage inside the battery. At the same time, ongoing hydrogen evolution changes the electrolyte’s pH, which further speeds up the deterioration of the battery system. Additionally, increased local pH causes the buildup of passivation products such as Zn_4_SO_4_(OH)_6_·*n*H_2_O or Zn­(OH)_2._ Passivation negatively impacts battery performance in two main ways: it consumes active materials, lowering Coulombic efficiency and long-term cycling stability, and it also raises internal resistance, which slows down the movement of both electrons and ions.^34,37,41^

### 3.2 Challenges of the Cathode Side

The dissolution of the cathode materials is most commonly studied in vanadium- and manganese-based oxides, which is one of the major challenges in ZIBs.^42,43^ The dissolution of cathode materials causes capacity degradation due to the loss of active material from the system. Additionally, the dissolved metal ions can redeposit on the anode, forming layers that lead to significant passivation.^44^

Divalent Zn^2+^ enables high capacity and energy density through multielectron transfer; its high charge density creates strong electrostatic interactions within cathode materials, expands interlayer spacing, and destabilizes the structure. Additionally, sluggish Zn^2+^ diffusion causes ion accumulation in the lattice, triggering irreversible phase changes in the cathode. For example, in vanadium-based cathodes, a common byproduct is Zn_3_V_2_O_7_(OH)_2_·2H_2_O, formed through strong Zn^2+^ interactions with vanadium–oxygen layers and reactions with water. Because Zn^2+^ is difficult to extract from its stable coordination, this phase is electrochemically inactive, leading to capacity degradation.^45^ Although initially reversible, it becomes irreversible with cycling due to trapped Zn^2+^ and water, contributing to capacity fading, a behavior also observed in Prussian blue analogs and manganese-based materials.^46,47^

To mitigate the above-mentioned challenges in both anode and cathode surfaces, electrode coating holds great significance in stabilizing the host structure and can provide a protective active material for battery systems. In this direction, ALD exhibits great potential in practical battery applications, as it can provide a uniform coating layer over the electrode at the atomic scale, even on a nanostructured 3D surface.^27^ In recent years, ALD has been widely used to apply thin protective coatings on electrode surfaces, limiting direct contact with the electrolyte and enhancing performance in Li- and Na-based batteries.^24,25^ Even ultrathin coatings, only a few nanometers thick, can significantly improve battery stability without adding noticeable weight.

## 4 Principles of Atomic Layer Deposition

In the early 1970s, ALD technology was called atomic layer epitaxy, and it was utilized for the fabrication of polycrystalline ZnS for thin film electroluminescent display.^23,48–50^ However, in the early 2000s, the sequential and self-limiting reactions on the surface were initiated. In this process, the growth of the precursor materials does not take place epitaxially on their underlying substrates. Therefore, it began to be known as ALD technology.^23,48–50^ The ALD technique is basically a special modification of the CVD process, because in the ALD process, the film growth occurs in a cyclic way. Typically, one growth cycle is composed of four stages, which include (1) the exposure of the first precursor material, (2) the purging of the reaction chamber, (3) second precursor material exposure, and (4) again, purging of the reaction chamber. This growth cycle process kept going continuously until the desired thickness of the films was achieved. Depending on the precursor materials and process utilized, each cycle can take between 0.5 s and a few seconds, which may result in deposition of the film thickness ranging in angstroms (Å).^18,27,48–50^

Each deposition cycle time variation is dependent specifically on the aggressiveness of the film fabrication reaction. The thickness of the film achieved in each cycle may be dependent on the precursor material molecules’ size. This is because of steric hindrance among larger-sized precursor materials, which limits the number of molecules that can adsorb on the substrate surface. To create a monolayer deposition, it is possible with a smaller size of element or molecule as precursor material.^18,27,48–50^ One more factor that influences the adsorption of the precursor material molecule is the number of adsorption-active sites on the surface. Under ideal conditions, after the completion of each exposure and purging step, the reacted or chemisorbed precursor molecule with the surface groups saturates. When the chemisorbed layer is formed, no additional adsorption occurs on the surface. Thus, the ALD deposited film growth is self-limiting.^18,48–50^ Therefore, to utilize the self-limiting and conformal thin layer properties of the ALD technology, our group utilized a combination of 3D printing technology with the ALD technique to enhance the electrochemical properties of stainless steel (SS) electrodes, by coating TiO_2_ layers, which can be used for multiple electrochemical applications (Figure 2a).^27^ Herein, the ALD deposition process can be carried out as described in Figure 2b. The first cycle involves the pulsing of the precursor materials TiCl_4_ for about 0.2 s, followed by the purging of N_2_ gas in the ALD chamber to evacuate any unabsorbed precursor materials onto the substrate. Later, the H_2_O reactant was pulsed for 0.5 s and in the end again purged with N_2_ gas in the ALD chamber.^27^ Afterward, different samples by varying the number of cycles of TiO_2_ layers can be grown onto the different substrates tailored for specific electrochemical applications.^27^

**Figure 2:**
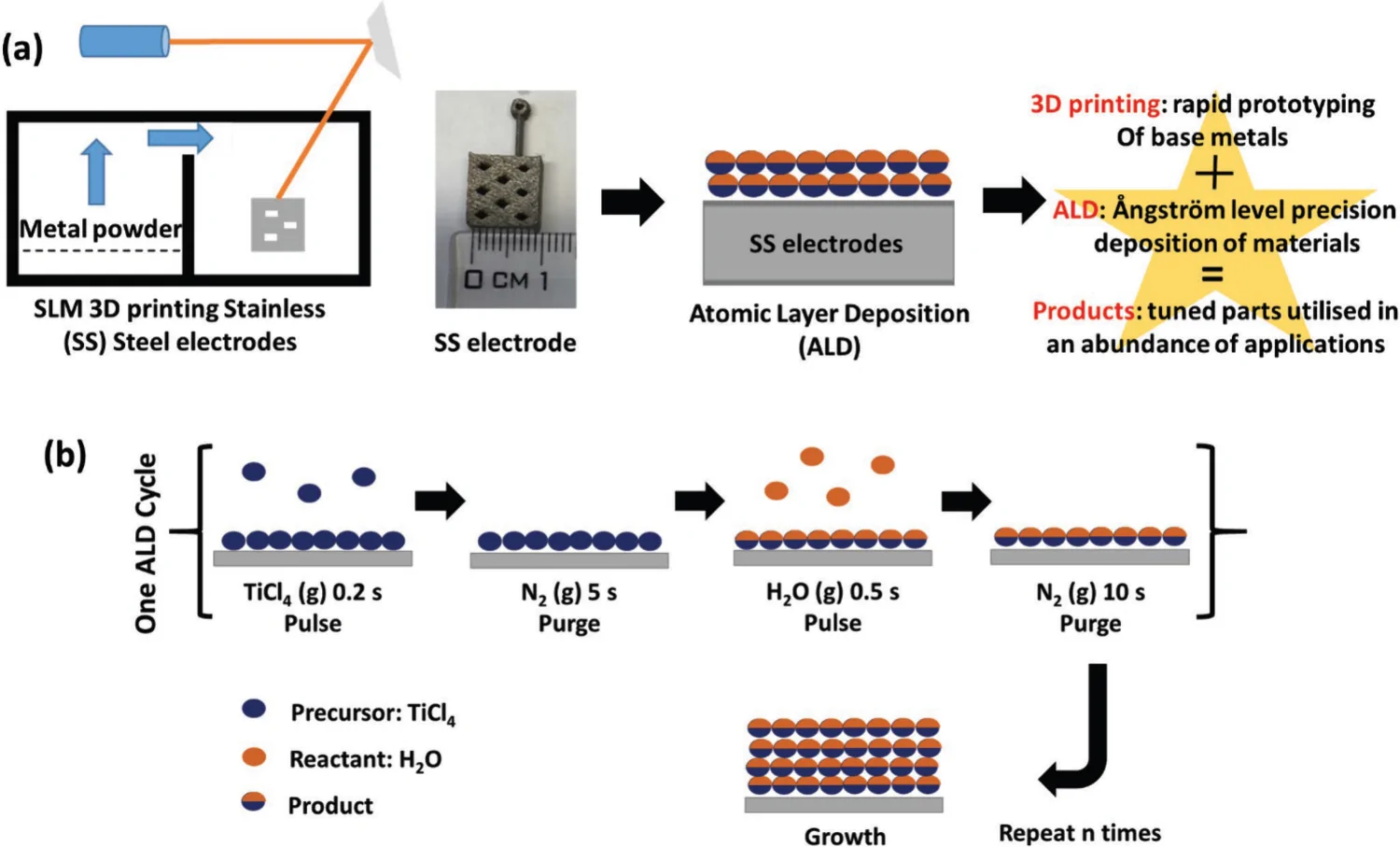
(a) The synthesis method of the 3D printed stainless steel electrodes, followed by ALD coating on the 3D printed stainless steel electrodes. (b) A scheme demonstrating the ALD deposition process. Reproduced with permission from ref 27. Copyright 2019 Wiley-VCH.

Moreover, to better understand the thickness, morphology/growth, and structure of the grown TiO_2_ layers onto the substrate, Browne et al. and colleagues demonstrated that the TiO_2_ layers can be uniformly grown on the surface of silicon wafers that have a smoother surface compared to the 3D printed stainless steel surface. It is easier to understand the growth of TiO_2_ layers deposited via ALD on the smoother surfaces. Subsequently, to confirm that the varying number of ALD cycles varies the morphology and growth of the TiO_2_ layers onto the substrate, scanning electron microscopy (SEM) images were displayed (Figure 2).^27^ The structural morphology of the TiO_2_ layers after 400 cycles, as shown in Figure 3a, resembles that of the blank substrate. Nonetheless, the morphology alters after the number of cycles is increased to 800 and 1200 ALD cycles, confirmed by SEM images, which show TiO_2_ grains with discernible boundaries, indicating successful formation of thin films (Figure 3b,c). In summary, this work demonstrates that ALD technology can be integrated with 3D printing technology to create functional electrodes for electrochemical applications.^27^

**Figure 3:**
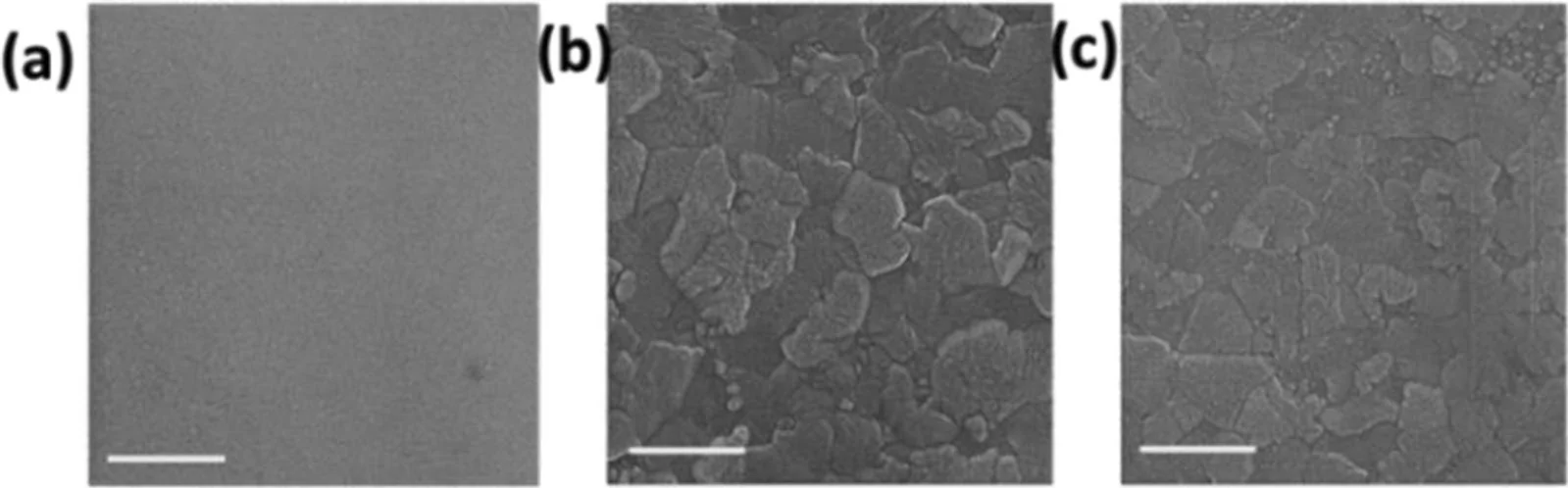
Growth of TiO_2_ via ALD on the Si wafer surface and their corresponding SEM micrographs at different cycle numbers: (a) 400, (b) 800, and (c) 1200 (scale bar = 500 nm). Reproduced with permission from ref 27. Copyright 2019 Wiley-VCH.

### 4.1 Advantages of ALD in ZIBs

ALD is an innovative advanced technology to create a precise, uniform ultrathin coating at the atomic scale level on the complex electrode surface, which could offer several benefits for the enhancement of the ZIB performance, durability, and longevity. Nanoscale engineering to prevent cathode dissolution suppresses dendrite growth, which leads to an increase in the electrochemical performance with customizable layer properties like thickness, compositions, and functionality.^51–60^ Further, in this section, we will discuss some recent studies to modify cathode and anode electrodes for ZIBs using the ALD technique, which explains the benefits of ALD for ZIBs and resolves the challenges mentioned above related to ZIBs.

### 4.2 Cathode Modification Using ALD for ZIBs

Vanadium-based oxides are widely explored as cathode electrodes in ZIBs. However, several challenges persist in the designing and development of vanadium-based materials, which include V dissolution, irreversible phase transition, and sluggish Zn^2+^ diffusion reaction kinetics. To address this issue, Hu et al. formulated a cathode material using a two-step synthesis process known as ZVO@MoS_2_ core–shell heterostructures. First, by employing an ultrafast and colloidal chemical fabrication process, they synthesized a crystalline Zn_0.25_V_2_O_5_ (*c*-ZVO), followed by the ALD technique to coat the surface of ZVO with uniform MoS_2_ layers to fabricate ZVO@MoS_2_ core–shell heterostructures.^51^

The successful formation of the ZVO@MoS_2_ core–shell heterostructures is confirmed by XRD and morphological characterization, as illustrated in Figure 4a–h. The atomic-layer-precise engineered atomic-layer-deposited MoS_2_ with the semimetallic 1T′ phase serves as the effective artificial cathode electrolyte interphase. The theoretical calculations displayed the energy needed for Zn^2+^ ions’ (de)­intercalation in the *a*-ZVO@MoS_2_ is −1.41 eV, which is lower compared to the *c*-ZVO, i.e. −2.69 eV, which further validates the experimental findings confirming the nanoscale engineered atomic-layer deposited 1T′-MoS_2_ phase increases the conductivity, prevents the V-dissolution of ZVO, and maintains stable reversible capacity (Figure 4i and 4j).^51^

**Figure 4:**
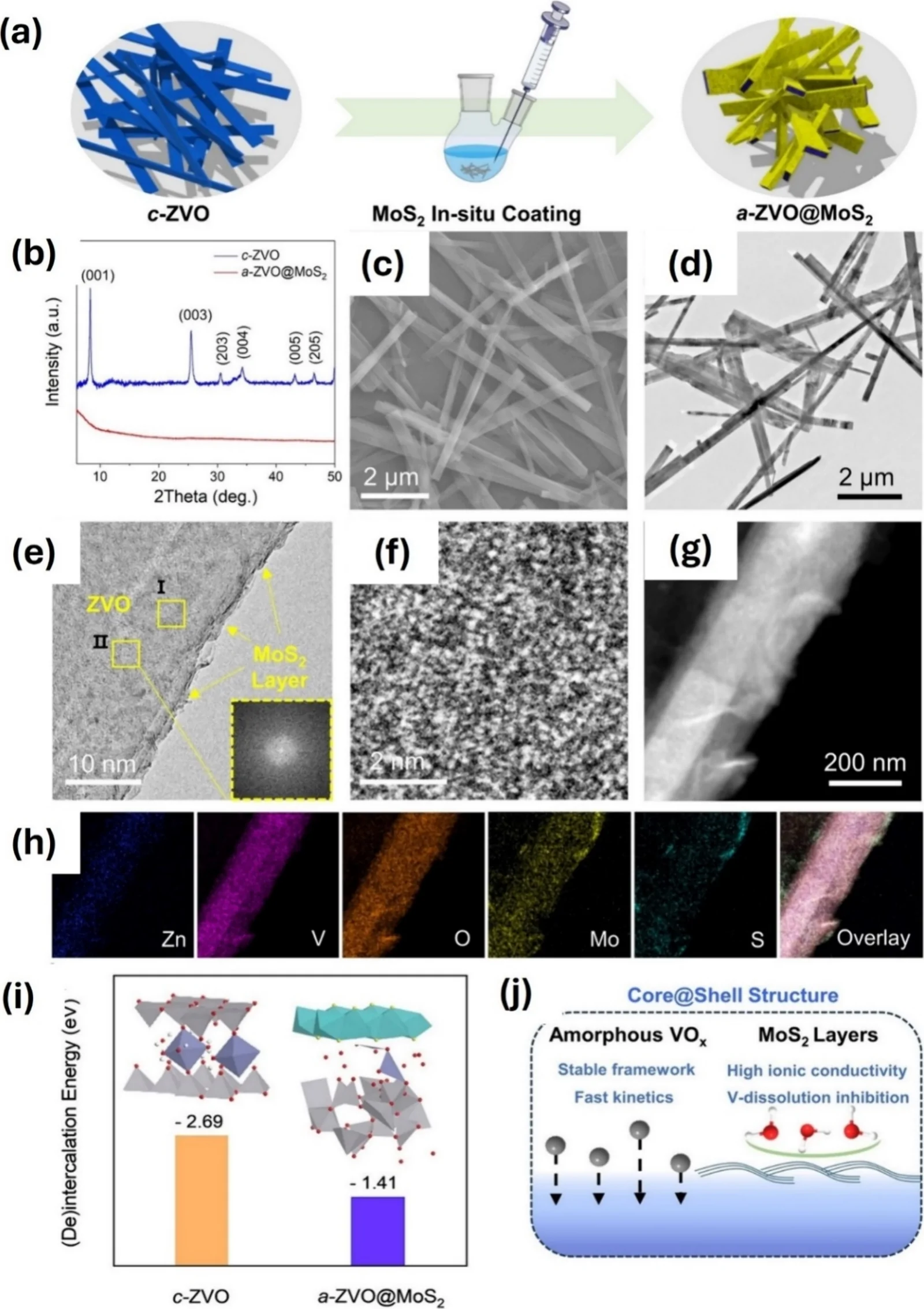
ALD-modified cathode for ZIBs. (a) Schematic of the fabrication and characterization of *a*-ZVO@MoS_2_. (b) XRD graph, (c) SEM, and (d, e) TEM micrographs. Inset: FFT pattern, II region. (f) HRTEM of region I. (g) HAADF-STEM micrograph and (h) STEM-EDS elemental mappings. (i) The model of *a*-ZVO@MoS_2_ and *c*-ZVO Zn^2+^ and their (de)­intercalation energy. (j) Diagrammatic illustration of synergistic improvement through the *a*-ZVO@MoS_2_ structure. Reproduced with permission from ref 51. Copyright 2024 Wiley-VCH.

The as-fabricated ZVO@MoS_2_ cathode exhibited an excellent capacity of 293 mAh g^–1^, and after 300 cycles it displayed an extraordinary long cyclic stability of 75% with an ultralow current density of 0.05 A g^–1^.^51^ Further, Pumera et al. reported a 3D-printed-V_2_O_5_ cathode electrode Figure 5a, which provides a unique model for a scalable and tailored electrode for aqueous rechargeable ZIBs.^21^ In this work, they synthesized a hierarchical core–shell-type structure of a 3D-printed-V_2_O_5_ cathode electrode via ALD deposition of vanadium oxide on the surface of the 3D-printed nanocarbon frameworks. Bare 3D printed electrodes and ALD deposited V_2_O_5_ 3D-printed electrodes SEM images were obtained, confirming successful deposition of V_2_O_5_ (Figure 5b−e). Next, energy dispersive X-ray spectroscopy elemental analysis with SEM images was acquired (Figure 5f–i), which further confirms the presence of the uniform coating and distribution of V, along with the O and C atoms, suggesting V_2_O_5_ deposition.^21^ Additionally, Shuai et al. reported the deposition of a ZnO layer via the ALD technique on the cathode electrode (hydrated vanadium dioxide nanosheet array), which prevents V-dissolution and suppresses the side reaction on the surface of the cathode, increasing the overall ZIB performance. After 990 cycles, the cyclic stability of ZnO@VOH cathodes at a high current density of 5 A g^–1^ displayed improved capacity retention from 29% to 71%.^52^

**Figure 5:**
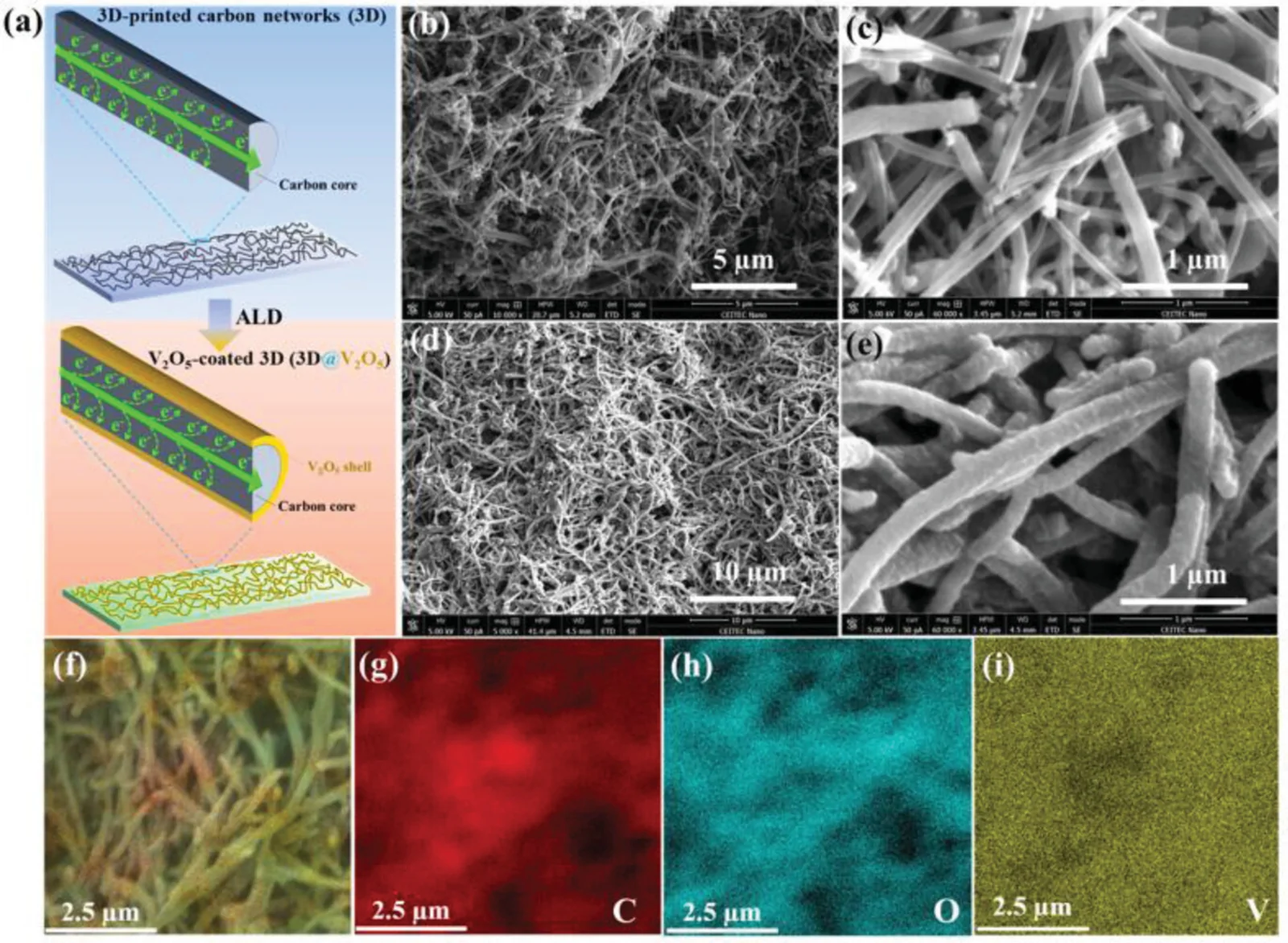
(a) Diagram demonstrating the core–shell structure of the 3D-printed@V_2_O_5_ electrode preparation process. (b and c) Before SEM micrographs of DMF-activated 3D-printed electrodes, carbon framework. (d and e) After 300 cycles of ALD deposition of V_2_O_5_. (f–i) Their elemental mapping. Reproduced with permission from ref 21. Copyright 2022 Wiley-VCH.

Moreover, Wu et al. reported aluminum-intercalated vanadate nanobelts on porous conductive carbon paper by tuning its surface hydrophilicity. A chemical-resistant passive layer of HfO_2_ with a thickness of 3 nm is deposited employing the ALD technique to avoid the corrosion of Al^3+^-ion-intercalated vanadates. This ALD deposition of HfO_2_ leads to enhanced overall electrochemical performance, providing cyclic stability up to 1000 cycles with 88% retention.^53^ Another study to prevent V dissolution and improve the stability of the cathode materials was reported by Yang et al. They deposited an Al_2_O_3_ layer using the ALD technique to boost the electrochemical activity of the vanadium diselenide nanosheet cathode materials.^54^ Zhang et al. reported a ZnO ultrathin coating through the ALD technique on the MnO cathode to enhance its electrochemical performance. The heterogeneous interface between ZnO and MnO creates an intense built-in electric field, which aids with the ion diffusion kinetics and increases the electron transport. Moreover, the ZnO thin layer efficiently reduces the MnO dissolution and improves its electrical conductivity.^55^

### 4.3 Anode Modification Using ALD for ZIBs

The ALD deposition of Al_2_O_3_ on the Zn anode was first reported by He et al. to improve the ZIBs’ performance. They assembled a Zn-MnO battery, where layered MnO_2_ serves as the cathode electrode and Zn serves as the anode electrode. The ultrathin Al_2_O_3_ (approximately 10 nm) coating on the Zn plate employing the ALD technique reduced the reactivity of the Zn anode with the electrolytic solution, causing passivation, enhancing the uniformity in the Zn plating/stripping, and reducing the formation of Zn dendrites. As a result, highly stable Zn metal anodes are engineered that improve the electrochemical performance of ZIBs, delivering a capacity retention of 89.4% after 1000 cycles at a current density of 1 mA cm^–2^ (3.33C for MnO_2_).^56^

Zeng et al. moved one step ahead and assembled battery devices with three different metallic oxide layers, such as Al_2_O_3_, TiO_2_, and Fe_2_O_3_, employing the ALD technique and exploring the relationship between the metal oxides and the crystallographic orientation surfaces in the ZIBs’ electrochemical performance. They concluded that the nucleation behavior of the (002) facet plays a crucial part in the preservation of a stable voltage profile and reversible lifespan. The Al_2_O_3_ exhibited a single function, which restricts the stripping process, resulting in a higher overpotential. In contrast, TiO_2_ and Fe_2_O_3_ tend to show multifunctionalities, preventing the influence of the electrolyte, stripping all other orientations, and protecting the (002) facet. The Al_2_O_3_@Zn displayed a (101) orientation; as a result, a random hexagonal hillock texture formed, and TiO_2_@Zn and Fe_2_O_3_@Zn obtained a smooth terrace texture.^57^

Moreover, the electrochemical characterization confirmed that the Fe_2_O_3_@Zn at a current density of 1 mA cm^–2^ delivered a minimal voltage hysteresis of 23.6 mV and the lowest overpotential of 31.64 mV. Additionally, among all the metal oxides, ALD layers of the Fe_2_O_3_@Zn tend to be more favorable for the diffusion of Zn^2+^ ions in the acidic media.^57^

Recently, a unique synergistic influence between the TiO_2_ layer, which is prepared by ALD, and the carbon nanotube (3D) network was studied. As a result, on the Zn anode, the 3D network morphology of CNT can assist with the abundant deposition active sites that boost the reaction kinetics and lower the nucleation overpotential. Meanwhile, the even deposition of the Zn was repetitively supported by the TiO_2_ layer due to its outstanding zincophilic nature.^58^ To further advance the ALD technique, a multifunctional protective layer for highly stable Zn anodes was reported, where atomic Sn metals as active sites were anchored on nitrogen-doped carbon (NC), where NC serves as support.^59^

The Sn–N–C layer serves as a hydrophobic protective layer to prevent water permeation, which results in suppressing the hydrogen evolution reaction, and it also guides Zn nucleation due to their zincophilic sites, which leads to uniform electron distribution assisted by the charge transfer by conductive NC. The hydrophobic/hydrophilic bilayer organization of the porous Sn-NC leads to a rapid Zn^2+^ ion diffusion route. As a result, cyclic stability of over 280 h at current density 10 mA h cm^–2^ and a low nucleation overpotential of 7.5 mV are accomplished in the symmetric cells.^59^ Moreover, a simple, facile, and efficient way to modify anode and cathode electrodes to assemble excellent performing ZIBs was reported, where the sonochemical technique is employed to fabricate ammonium vanadate nanofibers (AVNFs) intercalated with NH_4_^+^ and H_2_O, which serve as a cathode (Figure 6a). Additionally, an ultrathin Al_2_O_3_ with a thickness of 9.9 nm was deposited by ALD on the Zn anode electrode as illustrated in Figure 6b. The ALD coating minimizes the cell polarization, suppresses the dendrite growth during the cyclic process, and creates a stable ion route. The AVNF//60Al_2_O_3_@Zn device rate capability is 108 mA h g^–1^ at a current density 20 A g^–1^.^60^ To better understand how different varieties of materials for ALD coatings address each specific challenge in ZIB electrochemical performance and their advantages, we provided a comparative table in the Supporting Information as Table S1.

**Figure 6:**
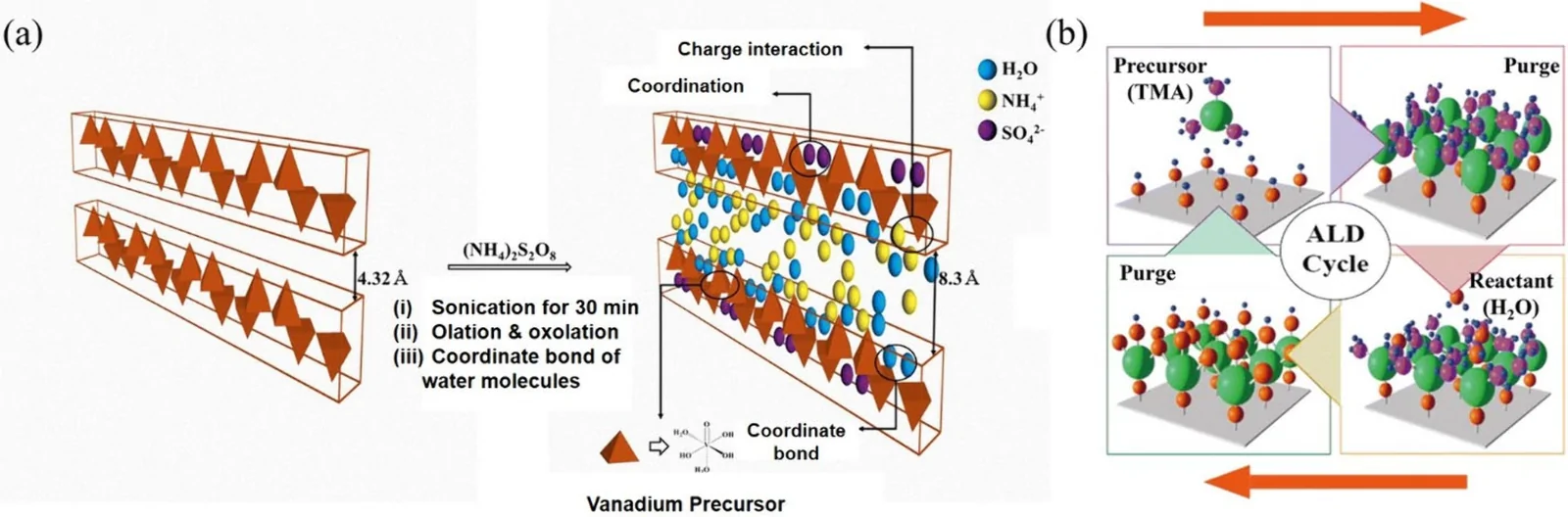
ALD-modified anode for ZIB. (a) Graphic of the formulation of an ammonium vanadate nanofiber (AVNF) cathode by employing the sonochemical method. (b) Schematic of ALD cycles to deposit Al_2_O_3_ on the Zn anode from its precursors. Reproduced with permission from ref 60. Copyright 2023 Royal Society of Chemistry under Creative Commons Attribution NonCommercial 3.0 Unported License.

## 5 Conclusion and Perspectives

In conclusion, massive attempts have been made in the advancement of the ALD technique, which has evolved as a promising candidate in high-performance ZIBs. As the ALD technique can deposit the materials on the surface of electrodes with ultrathin, uniform coatings and atomic-scale precision, ALD is a powerful tool to modify the cathode and anode electrode surface properties in ZIBs by fine-tuning the interface properties and nanoengineered surface. The exceptional features of the ALD technique enable the fabrication of an artificial interphase between electrodes and the electrolyte, generate protective layers, and modify the surface properties, which can substantially boost the electrochemical performance, cycling life, stability of electrode materials, and safety of ZIBs.^51–60^

ALD is an extremely versatile and innovative technique that enables users to design uniform thin layers with customized material thickness and composition on a variety of different surfaces. Metal oxide coatings on the different types of surfaces for both the cathode and anode have been generated using ALD technology. These metal oxide layers have significantly improved the electrochemical performance by suppressing the growth of dendrites, reducing side reactions like HER and corrosion, and limiting the electrode–electrolyte interaction on the anode electrodes. On the cathode electrode, the ALD coating of a metallic layer reduces the material dissolution for vanadium- and manganese-based oxide cathodes, which leads to structural stability and improves the cathode material’s electrochemical performance.^51–60^

However, there are still a few obstacles that need to be addressed before the implementation of ALD technology for the mass-scale commercial production of ZIB devices. A few major issues with ALD include the expenses associated with the ALD technology, relatively slow deposition rate, which impedes mass-scale manufacturing, and the selection of suitable precursor materials and deposition conditions that should be precisely controlled to regulate the integrity of the electrode materials.

In the future direction, ALD is at the forefront of nanoscale engineering technology for modulating the surface properties and boosting the electrochemical performance of ZIBs. By resolving the existing limitations, we can exploit its atomic-level precision and uniformity. ALD is an innovative cutting-edge technology that has the potential to play a key role in the advancement of sustainable and safe ZIB devices for next-generation energy applications. One of the promising directions could be the exploration of different kinds of materials, such as metal phosphides and metal nitrides, for ALD technologies. Until now, the most explored precursor materials have been made up of different types of metal oxides.

Another fascinating direction could be the design and development of novel materials with desirable dimensions and morphologies at varying scales. ALD provides much better regulation in the material growth. Additionally, by employing ALD technology, materials could be tailored for specific electrochemical applications, which would lead to boosting the electrochemical performance and would also assist in a better understanding of the basic chemistry of materials. Further, the advancement of ALD can be possible by the fabrication of materials with a combination of different technologies, such as laser processing and advanced printing technology, for the synthesis of different nanomaterials. Additionally, as progress continues in the ALD technology, discovering materials with low cost and sustainability should be the priority (Figure 7).

**Figure 7:**
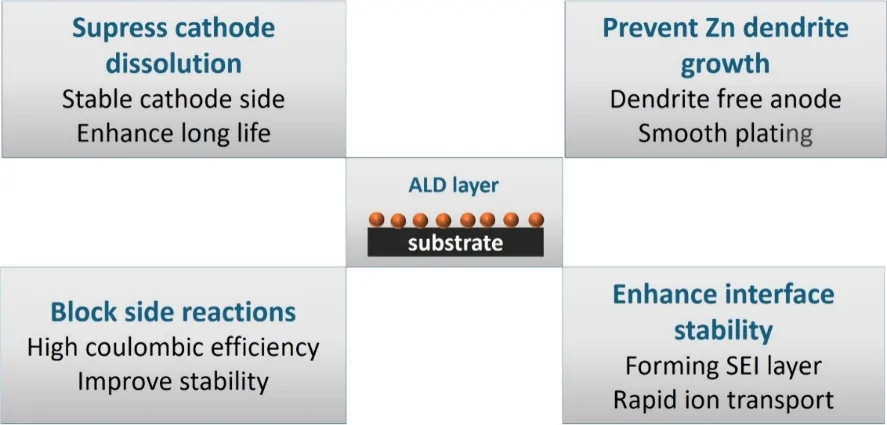
Overall ALD coating benefits for ZIBs.

## Supplementary Material



## Notes

Supporting Table S1: Comparative table of different types of materials, ALD coatings for specific ZIB challenges, and their electrochemical performance and advantages (PDF)
