# Supplementary material for: Atomic Layer Deposition for Zinc-Ion Batteries: Nanoarchitectonics for Next-Generation Energy Solution
Source: Acc Mater Res. 2026 Jun 25;7(8):832–43. doi: 10.1021/accountsmr.5c00326 (PMC13533321; doi:10.1021/accountsmr.5c00326)
Supplement: Supplementary file 1 [file mr5c00326_si_001.pdf]

Supporting information (SI)

Atomic Layer Deposition for Zinc-Ion Batteries: Nanoarchitectonics for Next-Generation Energy Solution

Shaista Nouseen [a,b], Sujit Deshmukh [c], Martin Pumera [a,b,d,e*]

[a]*Quantum Materials Laboratory, 3D Printing & Innovation Hub, Center for Nanorobotics and Machine Intelligence, Department of Chemistry and Biochemistry, Mendel University in Brno, Zemedelska 1, Brno 61300, Czech Republic*

[b]*Future Energy and Innovation Laboratory, Central European Institute of Technology, Brno University of Technology, Purkynova 123, Brno 61200, Czech Republic*

[c]*Department of Condensed Matter and Materials Physics, S. N. Bose National Centre for Basic Sciences, Block JD, Sector III, Salt Lake, Kolkata 700106, India.*

[d]*Advanced Nanorobots and Multiscale Robotics Laboratory, Faculty of Electrical Engineering and Computer Science, VSB - Technical University of Ostrava, 17. listopadu 2172/15, Ostrava 70800, Czech Republic*

[e]*Department of Chemical and Biomolecular Engineering, Yonsei University, 50 Yonsei-ro,Seodaemun-gu, Seoul, 03722, Korea*

Corresponding Author *Email: pumera.research@gmail.com, Martin Pumera

Table S1. Comparative table of different types of materials ALD coatings for specific ZIB challenge, their electrochemical performance and advantages.

**ALD coatings**	**Type of material coatings**	**Electrode modified**	**Zinc-ion Batteries challenge**	**Electrochemical performance**	**ALD-modification advantages**	**Type of ZIB**	**Ref.**
Metal oxide type	MoS_2	Cathode a-ZVO@ MoS_2 core@ shell heterostructures	V-dissolution and Sluggish Zn_2^+ diffusion kinetics	High capacity of 293 $mAhg^{-1}$, long-cycle durability of 75% after 300 cycles at current density of 0.05 A g^{-1}	MoS_2 with semi metallic 1T' phase improved electron transport and inhibits the V-dissolution	Aqueous zinc-ion batteries	[1]
	HfO_2	Cathode-HfO_2-coated-$Zn_3V_2O_7(OH)_2\cdot 2H_2O$ (ZVO)	Suppress surface reactions and Cathode dissolution	Capacity and cycle life (227 mAh g^{-1}@100 mA g^{-1}, 90% retention over 100 cycles	HfO_2 film as an artificial solid electrolyte interphase (SEI) decreases byproduct	Aqueous zinc-ion batteries	[2]

					and suppresses cathode dissolution		
	HfO_2	Cathode-AVO Nanobelts on C-Paper-HfO_2	Sluggish kinetics of Zn ion insertion	Cycling stability 88% retention at 1000 cycle	Prevent cathode degradation	Aqueous zinc-ion batteries	3
	Al_2O_3	Cathode-Vanadium diselenide nanosheets@N-CNFs (Al 2 O 3 @VSe 2 NSs@N-CNFs)	Cathode dissolution and structural instability of active materials	Energy density of ∼125 Wh kg − 1 , ultrahigh rate capability of 65.2% capacity retention after 200-fold increase in current density and 86.2% retention after 2500 cycles	Provides mechanical robustness in the cross-2D VSe 2@N-CNFs	Quasi-solid-state aqueous zinc-ion batteries (QAZIBs)	4

	ZnO	Cathode-ZnO@VOH	Vanadium dissolution and side reactions	Capacity retentions from 74 to 89% after 100 cycles at 0.5 A g^{-1} and from 29 to 71% after 990 cycles at 5 A g^{-1}.	Suppresses vanadium dissolution and side reactions	Aqueous zinc-ion batteries	5
	ZnO	Cathode-ZnO-coated MnO (MnO@ZnO)	Low electron conductivity and sluggish ions diffusion kinetics	Specific capacity of 350 mA h g^{-1} at 0.1 A g^{-1} and excellent long-term stability	Alleviate Mn-dissolution and also boost electrical conductivity	Aqueous zinc-ion batteries	6
	V_2O_5	Cathode-hierarchical core–shell structured cathodes	sluggish Zn_2^+ diffusion kinetics	Specific capacity (425 mAh g^{-1} at 0.3 A g^{-1}), energy and power densities (316 Wh Kg^{-1} at 213 W kg^{-1} and 163 Wh Kg^{-1} at 3400 W kg^{-1}), and rate	V_2O_5 offers abundant active sites and faster Zn2+ diffusion kinetics.	Aqueous rechargeable zinc-ion batteries	7

		$(3D@V_2O_5)$		performance (221 mAh g^{-1} at 4.8 A g^{-1}).		(ARZIBs	
	Al_2O_3	Anode-100Al_2O_3@Zn	Zn-dendrite growth, and hydrogen evolution problems	Reduced overpotential (36.5 mV) and cycling lifespan (over 500 h) at 1 mA cm-^{2} in Zn|Zn symmetrical cells	Wettability of Zn improved and corrosion inhibited	Aqueous zinc-ion batteries	8
	Fe_2O_3	Anode-150 cycles Fe_2O_3@Zn	Dendrites formation	Overpotential (31.64 mV) and minimal voltage hysteresis (23.6 mV) at 1 mA cm^{-2}	Fe_2O_3 layer tended to strip all orientations but the (002) facet	Aqueous zinc-ion batteries	9
	TiO_2	Anode-150 cycles TiO_2@Zn	Dendrite formation	Capacity starts from 172.2 to 227.8 mA h g^{-1} and reaches 192.6 mA h g^{-1} after 1000 cycles	TiO_2 layer tended to strip all orientations but the (002) facet	Aqueous zinc-ion batteries	9

	Al_2O_3	Anode-150 cycles Al_2O_3@Zn	Dendrite formation	Discharge capacity decreases from 148.1 to 147.2 mA h g^{-1} .	Al_2O_3 layer restrict stripping procedure	Aqueous zinc-ion batteries	9
	ZrO_2	Anode-400 cycles-40 nm ZrO_2@Zn,	Dendrite growth and side reactions	40 nm ZrO_2@Zn symmetry cell cyclic stability of 2200 h and 860 h at 0.5 and 10 mA cm^{-2}, respectively	Uniform Zn deposition, lowers the nucleation overpotential	Aqueous zinc-ion batteries	10
	TiO_2	Anode-100 times TiO_2–CNT@Zn	Dendrite growth	TiO_2–CNT@Zn high ultralong lifespan over 2000 h with a voltage hysteresis of 22.5 mV and Coulombic efficiency of 98.9% for more than 500 h.	Guide the uniform deposition of Zn due to excellent zincophilic nature	Aqueous zinc-ion batteries	11
	SnO_2	Anode-160-10 nm	Dendrite growth	SnO_2@Zn-MnO, specific capacity of 273 mAh g^{-1} at an	Reducing overpotential and	Aqueous zinc-ion	12

		SnO_2@Zn		imposed current rate of 100 mA g^{-1}	suppressing the hydrogen gas generation.	batteries	
	Al_2O_3	Anode-AVNF//60Al_2O_3@Zn	Dendrite growth	Rate capability 108 mA h g^{-1} even at 20 A g^{-1}, and ultra-high cycle stability with a capacity retention of 94% even after 5000 cycles at a current density of 10 A g^{-1}	Minimize cell polarization, create a stable ion pathway, and reduced dendrite formation	Aqueous zinc-ion batteries	[13]
Oxy-Nitride	Titanium oxynitride (TiN_xO_y)	Anode-(TiN_xO_y)@Zn-	Irregular dendritic growth, and corrosion.	cycling stability, maintaining performance over 1300 h at 1 mA cm^{-2} in a symmetric cell. TiN_xO_y)@Zn-MnO_2, capacity of 85.3 mAh g^{-1} after 4500 cycles at a current density of 10C	Reducing spontaneous corrosion and mitigating hydrogen evolution	Aqueous zinc-ion batteries	[14]

Atomic Sn sites nitrogen-doped carbon	Sn–N–C	Anode-Sn NC/Zn-	Anodic dendrites and side reaction	Lowered nucleation overpotential 7.5 mV, a steady low overpotential of ~23.8 mV at 1 mA cm^{-2}, and stable cycling (1 mA h cm^{-2} for 1000 h and 10 mA h cm^{-2} over 280 h.	Guide Zn nucleation at a nano-level due to rich atomic zincophilic sites and even electron distribution	Aqueous zinc-ion batteries	15

(1) Hu, C.; Li, B.; Nie, K.; Wang, Z.; Zhang, Y.; Yi, L.; Hao, X.; Zhang, H.; Chong, S.; Liu, Z.; Huang, W. Ultrafast Tailoring Amorphous $Zn_{0.25}V_2O_5$ with Precision-Engineered Artificial Atomic-Layer 1T′-MoS_2 Cathode Electrolyte Interphase for Advanced Aqueous Zinc-Ion Batteries. *Angew. Chem. Int. Ed.* **2025**, *64* (1), e202413173. https://doi.org/10.1002/anie.202413173.

(2) Guo, J.; Ming, J.; Lei, Y.; Zhang, W.; Xia, C.; Cui, Y.; Alshareef, H. N. Artificial Solid Electrolyte Interphase for Suppressing Surface Reactions and Cathode Dissolution in Aqueous Zinc Ion Batteries. *ACS Energy Lett.* **2019**, *4* (12), 2776–2781. https://doi.org/10.1021/acsenergylett.9b02029.

(3) Wu, H.; Ming, F.; Lei, Y.; Zhang, W.; Alshareef, H. N. Anisotropic Growth of Al-Intercalated Vanadate by Tuning Surface Hydrophilicity for High-Rate Zn-Ion Storage. *Small Struct.* **2020**, *1* (1), 2000040. https://doi.org/10.1002/sstr.202000040.

(4) Yang, J.; Yang, H.; Ye, C.; Li, T.; Chen, G.; Qiu, Y. Conformal Surface-Nanocoating Strategy to Boost High-Performance Film Cathodes for Flexible Zinc-Ion Batteries as an Amphibious Soft Robot. *Energy Storage Mater.* **2022**, *46*, 472–481. https://doi.org/10.1016/j.ensm.2022.01.014.

(5) Shuai, B.; Zhou, C.; Pi, Y.; Xu, X. Atomic Layer-Deposited ZnO Layer on Hydrated Vanadium Dioxide Cathodes against Vanadium Dissolution for Stable Zinc Ion Batteries. *ACS Appl. Energy Mater.* **2022**, *5* (5), 6139–6145. https://doi.org/10.1021/acsaem.2c00540.

(6) Zhang, G.; Xu, Y.; Wu, X.; Wang, J.; Li, W.; Li, X. Ultrathin ZnO Coating Layer to Boost the Electrochemical Reaction Kinetics of MnO Cathode for Advanced Aqueous Zinc-Ion Batteries. *Solid State Sci.* **2023**, *146*, 107371. https://doi.org/10.1016/j.solidstatesciences.2023.107371.

(7) Gao, W.; Michalička, J.; Pumera, M. Hierarchical Atomic Layer Deposited V_2O_5 on 3D Printed Nanocarbon Electrodes for High-Performance Aqueous Zinc-Ion Batteries. *Small* **2022**, *18* (1), 2105572. https://doi.org/10.1002/smll.202105572.

(8) He, H.; Tong, H.; Song, X.; Song, X.; Liu, J. Highly Stable Zn Metal Anodes Enabled by Atomic Layer Deposited Al_2O_3 Coating for Aqueous Zinc-Ion Batteries. *J. Mater. Chem. A* **2020**, *8* (16), 7836–7846. https://doi.org/10.1039/D0TA00748J.

(9) Zeng, Z.; Zeng, Y.; Sun, L.; Mi, H.; Deng, L.; Zhang, P.; Ren, X.; Li, Y. Long Cyclic Stability of Acidic Aqueous Zinc-Ion Batteries Achieved by Atomic Layer Deposition: The Effect of the Induced Orientation Growth of the Zn Anode. *Nanoscale* **2021**, *13* (28), 12223–12232. https://doi.org/10.1039/D1NR02620H.

(10) Liu, Y.; Guo, T.; Liu, Q.; Xiong, F.; Huang, M.; An, Y.; Wang, J.; An, Q.; Liu, C.; Mai, L. Ultrathin ZrO2 Coating Layer Regulates Zn Deposition and Raises Long-Life Performance of Aqueous Zn Batteries. *Mater. Today Energy* **2022**, *28*, 101056. https://doi.org/10.1016/j.mtener.2022.101056.

(11) Zeng, Y.; Wang, H.; Rauf, M.; Mi, H.; Sun, L.; Wu, Q.; Zhang, Q.; Ren, X.; Li, Y. Synergistic Enhanced Zinc-Ion Battery Performance Achieving by Atomic Layer Deposition of TiO2 on Three-Dimensional Carbon Nanotube Network Decorated Zn Anode. *Electrochim. Acta* **2023**, *447*, 142085. https://doi.org/10.1016/j.electacta.2023.142085.

(12) Gong, S. H.; Lim, H. J.; Lee, J. H.; Yoo, Y.; Yu, S.; Lim, H.-D.; Jung, H. W.; Ko, J. S.; Kim, I. S.; Kim, H.-S. Electrochemical Assessment of Highly Reversible SnO2–Coated Zn Metal Anodes Prepared via Atomic Layer Deposition for Aqueous Zn-Ion Batteries. *Appl. Surf. Sci.* **2023**, *611*, 155633. https://doi.org/10.1016/j.apsusc.2022.155633.

(13) Lee, S. H.; Han, J.; Cho, T. W.; Kim, G. H.; Yoo, Y. J.; Park, J.; Kim, Y. J.; Lee, E. J.; Lee, S.; Mhin, S.; Park, S. Y.; Yoo, J.; Lee, S.-H. Valid Design and Evaluation of Cathode and Anode Materials of Aqueous Zinc Ion Batteries with High-Rate Capability and Cycle Stability. *Nanoscale* **2023**, *15* (8), 3737–3748. https://doi.org/10.1039/D2NR06372G.

(14) Lei, P.; Liu, L.; Wang, X.; Su, Y.; Yan, K.; Wang, B.; Cheng, J. Ultrathin Surface Coating of Conductive and Zincophilic Titanium Oxynitride Enables Stable Zinc Anodes for Aqueous Zinc-Ion Batteries. *J. Colloid Interface Sci.* **2025**, *679*, 846–854. https://doi.org/10.1016/j.jcis.2024.09.240.

(15) Wang, Y.; Tan, Y.; Cheng, C. Atomic Sn Sites on Nitrogen-Doped Carbon as a Zincophilic and Hydrophobic Protection Layer for Stable Zn Anodes. *J. Mater. Chem. A* **2024**, *12* (1), 428–439. https://doi.org/10.1039/D3TA06372K.
